# A brief version of the Pediatric Inventory for Parents (PIP) in Spanish population: Stress of main family carers of chronic paediatric patients

**DOI:** 10.1371/journal.pone.0201390

**Published:** 2018-07-26

**Authors:** Sara Casaña-Granell, Laura Lacomba-Trejo, Selene Valero-Moreno, Vicente Prado-Gasco, Inmaculada Montoya-Castilla, Marián Pérez-Marín

**Affiliations:** Departament of Personality, Assessment and Psychological Treatments, Faculty of Psychology, University of Valencia, Valencia, Spain; TNO, NETHERLANDS

## Abstract

A chronic illness in childhood has a negative impact on the paediatric patient and on family functioning. Psychological stress in parents influences the level of adjustment to the illness of their children. The Pediatric Inventory for Parents (PIP) was designed to measure stress in parents whose child has a chronic illness or requires prolonged medical monitoring. The main objective of this study is to provide a brief version of the Spanish translation of the PIP, across a sample consisting of 465 main familial caregivers (85.2% female, n = 396) between 27 and 67 years old ($\bar{X}$ = 44.13; SD = 5.35) of paediatric patients between 9 and 18 years old ($\bar{X}$ = 12.10, SD = 2.20; 56.8% men, n = 264) diagnosed with diabetes mellitus type I (20.9% of the sample; n = 97), short stature (32.5% of the sample; n = 151), or a chronic respiratory disease (asthma, cystic fibrosis, bronchiolitis obliterans and bronchiectasis) (46.6% of the sample; n = 217). After performing several EFAs (Exploratory Factor Analyses) and CFAs (Confirmatory Factorial Analyses), it was decided that 30 items need to be removed. Reliability and validity results suggest that the new 12-item version possesses appropriate psychometric properties. Cronbach’s alpha value ranging between α = .42 and α = .81 and fit values obtained indicate a good fit: χ^2^/df (88.393/48) = 1.84 (α < .01); S-B χ^2^(df) = 88.393 (48); CFI = .95; IFI = .95; RMSEA = .05 (.033 - .074) for the frequency scales and χ^2^/df (72.002/48) = 1.5 (α < .01); S-Bχ^2^(df) = 72.002 (48); CFI = .97; IFI = .97; RMSEA = .04 (.011 - .063) for the difficulty scales. The PIP also showed predictive ability in regards to anxiety and depression, a positive relationship between the instrument's own scales and a negative relationship with the caregiver's age. Finally, depending on the paediatric patient's diagnosis, differences in stress levels were found.

## Introduction

Chronic childhood illnesses have a negative impact on the paediatric patient, on his or her parents, on family functioning and also on medical services [1–3]. Their incidence is increasing and they currently affect between 10% and 20% of children [4].

Various studies have shown a link between high levels of parental stress and the need to provide care to a chronically ill child [2,5]. Indeed, stress and emotional distress levels are higher for such parents than for caregivers of healthy children, with this difference being especially pronounced in the case of mothers [6]. Parents and children who have high levels of stress report greater levels of anxiety and depression [7–9] and an increase in family conflicts [10]. It is important to pay attention to the caregiver's adjustment to his or her child's diagnosis, as inappropriate parental behaviors have been observed to influence the patient's symptoms. [11].

The combination of the caregiver's fears and concerns, along with financial, social, emotional, and health problems—both physical and psychological—end up producing what is known as the caregiver's burden [12]. Zarit et al. [13] define the caregiver's burden as the set of attitudes and emotional reactions derived from the act of caregiving, as well as the degree of disturbance that occurs both in the home and in the caregiver's life in general. This overload may negative influence adherence to treatment and disease prognosis in the paediatric patient [14–16].

Despite the important role played by the caregiver, the healthcare system does not pay sufficient attention to the distress he or she experiences as a consequence [17]. New user-friendly tools should be developed for parents to help guide a more focused and personalized intervention wherever they may need more help.

To assess the stress levels of parents coping with the chronic illness of their child, Streisand et al [18] developed the Pediatric Inventory for Parents (PIP). Cousino and Hazen [5] reported that the PIP is a commonly-used measure of such stress. The PIP is based on Lazarus and Folkman’s [19] Transactional Model of Stress. The PIP examines 42 potentially stress-inducing situations for parents of children with a chronic illness and each item seeks to establish the difficulty of each situation and the frequency with which they occur. The items in the questionnaire are divided into four domains or subscales: medical care, communication, emotional functioning and role function. A frequency and difficulty score can be obtained for each subscale by adding together the score for each item it contains. Also, by adding together the different subscale scores, the overall frequency and difficulty scores can be obtained for the situations proposed.

This instrument has been used with parents of children with a variety of illnesses including: endocrinological pathologies such as diabetes mellitus type 1 (DM1) [20,21], short stature (SS) [22], and obesity [23]; chronic respiratory diseases [24]; cancer [25]; chronic pain [26]; blood disorders such as sickle-cell disease [27] and haemophilia [28]; gastrointestinal disorders [29]; congenital diseases [30]; mitochondrial diseases [31]; neuropsychological disorders [32]; and also those undergoing surgery [33,34]. However, it is common for studies to consider only a single pathology in their research, rather than analyzing several of them at the same time, and are therefore unable to determine diagnosis-specific differences.

The PIP has been translated into different languages such as Spanish [35], German [34] and Portuguese [36], and for use with family caregivers in different paediatric populations, such as those suffering from diabetes, short stature, haemophilia, cancer and weight problems [35, 22, 23, 34, 36, 37].

When it comes to the relationship between PIP scores and the caregiver’s age some discrepancies have emerged in the literature. Some authors indicate that there is none [35], others that younger parents report higher scores [18], and there are studies in which older parents are the ones who report higher levels of stress [38].

Despite its wide clinical use in different populations, the length of the original instrument makes it difficult to apply in clinical practice. In addition, the studies do not provide sufficiently robust empirical evidence on the psychometric properties of this instrument in those populations. Some of the deficiencies are: the use of small samples, the scale properties have not been subjected to a confirmatory factor analysis (CFA), symptoms of depression in the caregivers have not been taken into account, and a range of pathologies have not been considered in the same study.

For example, DM1, chronical respiratory disease and SS are three of the medical conditions that produce the most medical consultations and diagnoses in chronic paediatric care around the world, including Spain [39–43]. Caregivers of paediatric patients with any of these three diagnoses have shown elevated levels of stress [20–22, 24] although they have always been studied separately and there are no studies comparing the level of stress between these pathologies.

Given the importance of detecting caregiver stress to avoid the overload that is caused by care, it is paramount to facilitate the use of PIP in clinical practice. For this and the above reasons, the objective of this study is to provide a brief version of the validated Spanish translation and by Del Rincón, Remor and Arranz [35] of the Pediatric Inventory for Parents (PIP) across a sample of diverse pathologies (DM1, SS and chronic respiratory diseases), while using a robust methodology and seeking to ascertain their relationship with anxiety and depression (HADS) [44]. As secondary objectives, the relationship between PIP scores and caregiver age will be analysed, as well as whether there are differences in stress levels depending on the paediatric patient's diagnosis.

## Materials and methods

### Participants

The sample consisted of 465 main familial caregivers (85.2% female, n = 396) aged between 27 and 67 ($\bar{X}$ = 44.13; SD = 5.35), of paediatric patients between 9 and 18 years old ($\bar{X}$ = 12.10, SD = 2.20; 56.8% men, n = 264) diagnosed with DM1 (20.9% of the sample; n = 97), SS (32.5% of the sample; n = 151) or a chronic respiratory disease (asthma, cystic fibrosis, bronchiolitis obliterans, and bronchiectasis) (46.6% of the sample; n = 217), all of whom were treated in Valencia, Spain, at three of the region’s main hospitals. The only participants included in the study were those who agreed to participate and who were the caregivers of paediatric patients between 9 and 18 years old who had been diagnosed at least 6 months prior to the study. Caregivers were excluded if the paediatric patient had, in addition to one of the diagnoses mentioned above, a more serious pathology or if they suffered from cognitive difficulties.

### Procedure

The data from this cross-sectional study was collected at the participating hospitals between April 2015 and October 2017. After the paediatric patients’ medical appointment and after being introduced by the patients’ physician, a trained member of the research team spoke to the caregiver about voluntarily and anonymously participating in the study. After signing a written informed consent form, a set of questionnaires was given to the caregiver. The questionnaire was completed by the caregivers themselves in the waiting area over the course of 30 minutes. When they finished, they gave the questionnaire to the member of the research team. This research has been approved by the Ethics Committee from the University of Valencia Review Board (IRB) and conducted according to the principles expressed in the Declaration of Helsinki.

### Instruments

#### Pediatric Inventory for Parents (PIP) [18, 35]

The PIP is a questionnaire comprising 42 items, grouped into four domains or subscales. Its purpose is to measure the levels of stress suffered by parents caring for a child with a chronic disease. Responses are provided using a five-point Likert scale, ranging from 1 (“never/not at all”) to 5 (“very often/extremely”). Higher scores indicate higher levels of stress. Earlier studies have reported good psychometric performance by the PIP, with α values ranging from .70 to .96 for the total frequency and difficulty scales [18, 35, 22, 23, 36, 37]. In the present study we do not carry out a Spanish translation of the instrument, using instead the one done by Del Rincón, Remor and Arranz [35], our objective being to offer a brief version of it. Del Rincón, Remor and Arranz [35] carried out the adaptation of the instrument in two phases: the first phase consisted of the translation followed by the inverse translation of the questionnaire, and the second phase included the evaluation of the psychometric characteristics of the questionnaire [35].

#### Hospital Anxiety and Depression Scale (HADS) [44]

This is a screening tool that can detect emotional distress in non-psychiatric patients in a hospital setting. The questionnaire comprises 14 items grouped in two subscales, one for anxiety and one for depression, and a global one for emotional distress. Responses are given using a Likert scale ranging from 0 to 4. In a validation study in the Spanish population, adequate psychometric properties were found with the anxiety scale ranging from .68 to 0.93 (mean .83) and with the depression scale ranging from .67 to .90 (mean .82) [45].

### Data analyses

The data analysis was performed using SPSS 23.0, EQS 6.3 and FACTOR software [46].

Since the questionnaire is made up of two different scales, one for frequency and the other for difficulty, all of the analyses done have been carried out as if they were two different instruments (Frequency and Difficulty). Each of the procedures performed for these scales is described below.

First, as suggested by the literature [47,48], the properties of the items were analysed using observations of their mean, standard deviation, the item-total correlation coefficients and variations in the Cronbach's alpha coefficients if items were eliminated. Then, the psychometric properties were analysed using Cronbach’s alpha, exploratory factor analysis (EFA), and confirmatory factor analysis (CFA). According to recommendations found in the literature, the total sample (n = 465) was divided in two to perform EFA and CFA analyses [49]. Subsample A (n = 230) was used to conduct EFA and subsample B to conduct the CFA (n = 235). Each sample was selected by controlling or counterbalancing sampling based on age, sex, and diagnosis.

Before performing EFA and CFA, the suitability of the samples was assessed by means of a Kaiser-Meyer-Olkin (KMO) test and Barlett’s sphericity test. Values above 0.75 in the KMO test indicate that a factorial analysis is appropriate, while Barlett’s sphericity test must be statistically significant (p≤.05) [50].

After performing several EFAs and CFAs, in order to achieve a good fit of the model, it was necessary to remove 30 items. Consequently, the final instrument consisted of 12 items (each item required two responses: one for frequency and another for the difficulty it causes) distributed in 4 dimensions, as in the original instrument. The process for reaching such a solution is explained below.

The EFAs were calculated using FACTOR [46], according to the process recommended by Lloret-Segura et al. [49], using the unweighted least-squares method (ULS), the application of the method of parallel analysis, and the direct Oblimin rotation. First, the 42 items that make up the original questionnaire were considered, although parallel analysis suggests using only one dimension for each scale (Frequency and Difficulty), we decided to analyse the factorial solution with the theoretical four dimensions per scale (Frequency and Difficulty). Initially, all items on the scale were considered, but since the scales were not properly adjusted, items that saturate below .40 in one factor and/or saturate in more than one factor were removed. In addition, with the objective of extracting a reduced version of the same, it was decided to retain those items that presented greater saturation with the factor or latent variable, considering always having at least 3 items, indicators or variables observed by factor or latent variable as suggested by the literature [51,52]. All of which resulted in the elimination of 30 items from each scale, as was the case with the reliability analysis. The final structure consists of 12 items grouped into four dimensions. According to experts, an EFA model presents good fit when the Comparative Fit Index (CFI), and the Goodness of Fit Index (GFI) present values greater than .90 and the root mean-square error of approximation (RMSEA) exhibit values equal or lower than .08 [49,53].

After performing EFA, CFA was used to validate the factorial structure of the scales using maximum likelihood estimation (MLE) and the robust Satorra-Bentler adjustment to correct for the absence of multivariate normality [54]. The adequacy of the CFAs was tested using the significance of chi-squared and of the robust Satorra-Bentler correction (S-B χ^2^) [54–55]. Additional coefficients were calculated, such as the χ^2^ ratio and its degrees of freedom (χ^2^/df) as well as the S-B X^2^ and its degrees of freedom—with values of less than five being acceptable [56–57]—which made it possible to test the adequacy of the proposed models.

In addition, the coefficients of the robust indices of goodness of fit of the proposed models were tested considering the comparative fit index (CFI), and incremental fit fix (IFI). For these indicators, values greater than .90 were considered a good fit [58]. To conclude, the root mean-square error of approximation (RMSEA) was computed, these ratings were required to be less than .08 to be considered a good fit [53]. With regard to the models analysed—and the process of their re-specification that has been followed—first, as with the EFA, the original 42 items were grouped in the theoretical four dimensions (for each of the scales, Frequency and Difficulty). Afterwards, the reduced version of 12 items grouped into the 4 dimensions (12 items for each of the scales, Frequency and Difficulty) was tested.

In order to increase the empirical evidence for construct validity the convergent validity of the scale was calculated. To obtain convergent validity the scale items need to be significantly correlated with the latent variables that one would expect to be measured (T values >1.96) and with factor loading being above .70 [59].

After analysing the internal structure of the instrument, the relationships between the four dimensions of the instrument were analysed using Pearson correlation analysis. Subsequently, the instrument’s nomological or criterion validity was analysed by observing the relationships between disease adjustment (measured through PIP) and emotional distress, anxiety and depression (measured through HADS) using two differential methodologies, Pearson correlations and hierarchical regressions, in which the PIP dimensions acted as predictors of the HADS dimensions.

Finally mean differences in relation to diagnosis (ANOVA with Tukey post hoc) were then analysed.

## Results

### Reliability and item analyses

Table 1 shows the final list of items chosen after the modification of the original instrument. The table shows the items (the item number in the original scale is in partheses), the mean value for each ($\bar{x}$), the standard deviation (SD), the item-total correlation (r_jx_) and Cronbach’s alpha value if the item were eliminated (α-x). The contribution of each item to the instrument seems to be satisfactory. In general, the values showed sufficient internal consistency, both in the case of the total scales for frequency and difficulty (α = .78 and α = .81 respectively), and for each of the domains: *communication* (α = .66 for frequency and α = .64 for difficulty), *medical care* (α = .69 for frequency and y α = .54 for difficulty), *role function* (α = .42 for frequency and α = .63 for difficulty), and *emotional functioning* (α = .77 for frequency and α = .66 for difficulty). The elimination of any one of them would not improve the instrument’s reliability.

**Table 1:** Item and reliability analysis.

Items	Frequency				Difficulty			
	α^a^ = .78; CI^b^ = .75-.81 (Total Scale)				α^a^ = .81; CI^b^ = .78-.83 (Total Scale)			
	M^c^	SD^d^	r_jx_^e^	α.-x^f^	M^c^	SD^d^	r_jx_^e^	α.-x^f^
**Communication**	**α**^**a**^ **= .66**				**α**^**a**^ **= .64**			
Waiting for my child’s medical test results (7)	2.86	.90	.39	.70	1.66	.97	.32	.70
Talking to my child about his/her illness (32)	3.23	1.16	.53	.50	2.12	1.25	.55	.39
Talking to family members about my child’s illness (37)	2.86	1.10	.53	.49	2.00	1.17	.51	.46
**Medical Care**	**α**^**a**^ **= .69**				**α**^**a**^ **= .54**			
Taking charge of changes in my child’s daily treatment (28)	2.96	1.45	.46	.64	1.93	2.26	.27	.70
Helping my child with medical procedures (33)	3.50	1.42	.61	.43	1.92	1.32	.51	.28
Accompanying my child during medical tests and treatment (38)	4.10	1.26	.43	.67	1.97	1.37	.38	.42
**Role Function**	**α**^**a**^ **= .42**				**α**^**a**^ **= .63**			
Having little time to attend to my own needs (25)	3.11	1.23	.27	.33	2.60	1.27	.42	.56
Feeling uncertain about disciplining my child (35)	2.26	1.12	.37	.24	2.33	1.32	.44	.53
Noticing a change in my relationship with my partner (41)	2.17	2.23	.24	.53	2.25	1.31	.49	.51
**Emotional Functioning**	**α**^**a**^ **= .77**				**α**^**a**^ **= .66**			
Worrying about the long-term consequences of the disease (24)	3.48	1.12	.61	.68	3.12	1.96	.40	.73
Feeling helpless regarding my child’s situation (26)	2.72	1.26	.59	.71	2.82	1.42	.54	.50
Feeling uncertainty about the future (29)	3.08	1.26	.62	.66	2.91	1.38	.54	.50

^a^ Cronbach’s alpha value.

^b^ Confidence Interval of Cronbach’s alpha.

^c^ Mean (M).

^d^ Standard deviation (SD).

^e^ Item-total correlation (rjx).

^f^ Cronbach’s alpha if the item is eliminated (α.-x).

### Validity analysis

In order to analyse the validity of the scale, as already stated, its internal structure was firstly analysed using EFA and CFA, then the relations between the dimensions of the instrument were analysed, and finally, with the aim of testing the criterial or predictive validity, the relations between PIP and HAD were studied.

Firstly, the suitability of the samples was assessed by means of a Kaiser-Meyer-Olkin (KMO) test and Barlett’s sphericity test. Values above 0.75 in the KMO test indicate that a factorial analysis is appropriate, while Barlett’s sphericity test must be statistically significant (p≤.05) [50]. The results were adequate and enabled the EFA and the CFA to be performed (Table 2).

**Table 2:** Values of KMO test and Bartlett’s sphericity test for subsamples A and B.

Sample and PIP scale	KMO^a^	Bartlett’s sphericity		
		χ^2^^b^	Df^c^	*p*^d^
Subsample A. PIP Frequency	.84	833.5	66	≤.001
Subsample A. PIP Difficulty	.88	862.2	66	≤.001
Subsample B. PIP Frequency	.82	762.5	66	≤.001
Subsample B. PIP Difficulty	.83	827.0	66	≤.001

^a^ Value of Kaiser-Meyer-Olkin Test.

^b^ Bartlett’s Statistic.

^c^ Degrees of Freedom of Bartlett’s Sphericity Test.

^d^ *p* Value of Bartlett’s Sphericity Test.

Then, after performing EFAs and CFAs, 30 items were eliminated and the final instrument now consisted of 12 items (each item required two responses: one for Frequency and another for the Difficulty it causes) distributed in 4 dimensions (as in the original instrument), with three items per factor. These results are discussed below.

Regarding the EFA, the factorial solution of the 12 items grouped in four dimensions presented good fit indexes (RMSEA = .07; CFI = .98; GFI = .99 for Frequency and; RMSEA = .02; CFI = .99; GFI = .99 for Difficulty). The variance explained by the four factors was 62.61% (Frequency scale) and 65% (Difficulty scale).

Considering the CFA, Table 3 compares the adjustment indexes of the original instrument with those of the brief version. Based on the results observed, although the PIP in its original form does not present an adequate adjustment for any of the scales (Frequency and Difficulty), the indicators obtained for the reduced versions do show good fit.

**Table 3:** CFA fit indicators for PIP with 42 items (original) and our brief version of 12 items.

Model	S-B-χ^2^ ^a^	*Df* ^*b*^	*p*	S-B χ^2^/df ^c^	CFI ^d^	IFI ^e^	RMSEA ^f^
PIP Frequency-42 items	1575.4188	813	< .000	1.93	.66	.69	.06 (.058-.068)
PIP Difficulty-42 items	1502.3941	813	< .000	1.85	.76	.77	.06 (.055-.065)
PIP Frequency-12 items	88.393	48	< .01	1.84	.95	.95	.05 (.033-.074)
PIP Difficulty-12 items	72.002	48	< .01	1.50	.97	.97	.04 (.011-.063)

^a^ Satorra-Bentler scaled chi-squared.

^b^ Degrees of freedom.

^c^ Ratio between χ^2^ & Df.

^d^Comparative fit index.

^e^ Bollet’s fit index.

^f^ Root mean-square error of approximation and 90% confidence interval of RMSEA.

Hereafter, the convergent validity of the scale was calculated. The convergent validity was satisfactory, demonstrating a strong significant correlation between the scale items and the latent variables that it aims to measure, with t values above 3.291 in each case and with the mean factor loading being above .70, with no improvement when higher loadings were used [59]. The next step was to analyse the relationship between the instrument’s different domains by means of Pearson correlation (Table 4).

**Table 4:** Correlations between the different PIP domains.

*Correlations between the different PIP domains*	1	2	3	4	5	6	7	8
1.CF ^a^	-							
2.MCF ^b^	.10*	-						
3.RFF ^c^	.22**	.34**	-					
4.EFF ^d^	.31**	.33**	.49**	-				
5.CD ^e^	.23**	.53**	.45**	.50**	-			
6.MCD ^f^	.10*	1.00**	.34**	.33**	.50**	-		
7.RFD ^g^	.22**	.34**	1.00**	.49**	.45**	.34**	-	
8.EFD ^h^	.31**	.33**	.49**	1.00**	.50**	.33**	.49**	-

^a^ Communication Frequency.

^b^ Medical Care Frequency.

^c^ Role Function Frequency.

^d^ Emotional Functioning Frequency.

^e^ Communication Difficulty.

^f^ Medical Care Difficulty.

^g^ Role Function Difficulty.

^h^ Emotional Functioning Difficulty.

*p < .0.

**p < .01.

Statistically significant correlations (from low to strong positive ones) were found between all the domains. Three particularly high correlations can be observed in the table: the relationship between Medical Care Frequency and Medical Care Difficulty (*p*≤.01, r = 1.00), Role Functioning Frequency and Role Functioning Difficulty (*p*≤.01, r = 1.00), and finally Emotional Functioning Frequency and Emotional Functioning Difficulty (*p*≤.01, r = 1.00).

After that, in accordance with the literature, the instrument’s criterion or predictive validity was established by means of determining the relationship between the PIP and other constructs suggested by the literature. Pearson correlation coefficients and hierarchical regression analyses were sought between the different instrument domains (PIP) and the HADS (Table 5). In general, the correlation coefficients were positive and significantly ranged between low to moderate (*p*≤.01, r ranged between .17 and .51). The correlations between the PIP scales and age were negative and significantly (*p*≤.05 and *p*≤.01), except in the case of Medical Care and Role Function (both in Frequency and Difficulty), which were not significant.

**Table 5:** Correlations of the PIP with the HADS domains.

*Correlations of the PIP with the HADS domains*	HADS			AGE
	Anxiety	Depression	Emotional distress	
CF ^a^	.30**	.23**	.29**	-.16**
MCF ^b^	.17**	.25**	.23**	-.02
RFF ^c^	.46**	.43**	.49**	-.08
EFF ^d^	.41**	.39**	.44**	-.13**
CD ^f^	.30**	.32**	.33**	-.16**
MCD ^g^	.17**	.25**	.23**	-.02
RFD^h^	.46**	.43**	.49**	-.09
EFD ^i^	.41**	.39**	.44**	-.13**
Total Frequency	.49**	.45**	.51**	-.14**
Total Difficulty	.42**	.44**	.47**	-.10*

^a^ Communication Frequency.

^b^ Medical Care Frequency.

^c^ Role Function Frequency.

^d^ Emotional Functioning Frequency.

^e^ Communication Difficulty.

^f^ Medical Care Difficulty.

^g^ Role Function Difficulty.

^h^ Emotional Functioning Difficulty.

*p < .0.

**p < .01.

Continuing with the criterion validity, three hierarchical regression analyses were undertaken, with the anxiety, depression and overall emotional distress domains being the criterion variables, and the different PIP domains and the parent’s age being the predictor variables. The first step was to include the parent’s age, followed by all the frequency domains, and finally the difficulty domains. The main results of the final models were:

- Regarding the prediction of anxiety, the inclusion of the main caregiver’s age increased variance for anxiety by 2% (ΔR^2^ = .02, p≤.01). With the inclusion of the four PIP frequency domains, the model improved significantly by 26% (ΔR^2^ = .26, p≤.001), whereas the inclusion of the four difficulty domains did not produce a significant increase (ΔR^2^ = .13, p = .716). Specifically, after the final step, the domains that were able to predict anxiety were Communication Frequency (β = .15, *p* ≤. .001), Role Function Frequency (β = .32, *p* ≤ .001) and Emotional Problems Frequency (β = .19, *p* ≤ .001) in a positive direction, and only Age (β = -.14, *p* ≤. .01) in a negative direction.
- Regarding depression, the inclusion of the main caregiver’s age increased variance for anxiety by 1% (ΔR^2^ = .01, p≤.05). The inclusion of the four PIP frequency domains improved the model significantly by 23% (ΔR^2^ = .39, p≤.001), whereas the inclusion of the difficulty domains did not effect a significant increase (ΔR^2^ = .01, p = .209). Specifically, after the final step, the domains that were able to predict depression were Role Function Frequency (β = .29, *p* ≤ .001) and Emotional Problems Frequency (β = .17, *p* ≤ .001) in a positive direction, and only Age (β = -.10, *p* ≤. .05) and Communication Frequency (β = -.10, *p* ≤ .05) in a negative direction.
- Finally, the inclusion of the main carer’s age increased the variance of overall emotional distress by 2% (ΔR^2^ = .02, p≤.01). The inclusion of the four PIP frequency domains improved the model significantly by 30% (ΔR^2^ = .30, p≤.001), whereas the inclusion of the difficulty domains did not effect a significant increase (ΔR^2^ = .01, p = .642). Specifically, after the final step, the domains that were able to predict emotional distress were and Role Function Frequency (β = .34, *p* ≤ .001) and Emotional Problems Frequency (β = .12, *p* ≤ .001) in a positive direction, and only Age (*β* = -.14, *p* ≤ .01) and Communication Frequency (β = -.14, *p* ≤ .01) in a negative direction.

### Mean difference

Finally, differences in caregiver’s stress depending on the paediatric patient’s diagnosis were analysed by means of an ANOVA and Tukey *post hoc* were performed.

No differences have been observed in the Difficulty scale, but in the Frequency scale, there were differences in the Communication Frequency (F = 20.84; p≤.01) and Total Frequency (F = 19.84; p≤.01). In general, caregivers of patients diagnosed with diabetes were those with the highest levels of stress ($\bar{X}$ = 10.12, SD = 2.25 for Communication Frequency; $\bar{X}$ = 40.87, SD = 8.93 for Total Frequency), followed by respiratory problems ($\bar{X}$ = 8.93, SD = 2.18 for Communication Frequency; $\bar{X}$ = 35.57, SD = 7.59 for Total Frequency) and finally the patients with SS ($\bar{X}$ = 8.16, SD = 2.54 for Communication Frequency; $\bar{X}$ = 34.34, SD = 8.71 for Total Frequency).

## Discussion

As we have seen, a chronic disease in childhood has a negative impact both on the child and on his or her family [5], causing high levels of stress and emotional distress [2, 5–9]. It is important to keep this in mind because parents’ maladaptive reactions may lead to additional physical and psychological complications for the paediatric patient [60]. The PIP was designed to evaluate the parents’ adaptation to their child’s diagnosis, it has been translated into different languages (Spanish [35], German [34] and Portuguese [36]), and it is probably one of the best-known questionnaires in use in this area. This instrument has been studied in different countries and on different illnesses, but, until now, no study has analysed psychometric properties by robust methods nor using large samples in the Spanish context, nor comparing between different pathologies in the same study.

For this reason, to fill this gap, and given the potential advantages of evaluating the adjustment of caregivers quickly and easily during medical consultations, the objective of this study was to provide a brief version of the PIP by studying the caregivers of paediatric patients diagnosed with DM1, SS and chronic respiratory diseases as a group. The study also examined the relationship of PIP to anxiety and depression, to the age of the caregiver, and the differences in stress depending on the diagnosis.

To obtain a brief version with adequate psychometric properties, 30 items need to be deleted from each scale (Frequency and Difficulty). The final version presented here contains 12 items for each scale (Frequency and Difficulty) distributed across four factors, with three items per factor.

Both reliability and validation results suggest appropriate psychometric properties. The reliability results were adequate with a Cronbach’s alpha value of .78 for the total Frequency scale and one of .81 for the total Difficulty scale. The fit values obtained with EFA analysis presented good fit indexes (RMSEA = .07; CFI = .98; GFI = .99 for Frequency and; RMSEA = .02; CFI = .99; GFI = .99 for Difficulty). The variance explained by the four factors was 62.61% (Frequency scale) and 65% (Difficulty scale). The fit values obtained with CFA analysis indicate that the model proposed seems to have a good fit: χ^2^/df (88.393/48) = 1.84 (α < .01); S-B χ^2^(df) = 88.393 (48); CFI = .95; IFI = .95; RMSEA = .05 (.033 - .074) for the frequency scales and χ^2^/df (72.002/48) = 1.5 (α < .01); S-Bχ^2^(df) = 72.002 (48); CFI = .97; IFI = .97; RMSEA = .04 (.011 - .063) for the difficulty scales.

As the literature suggests, PIP scores are also related to anxiety, depression, and overall emotional distress [7–9]. Our results point to a positive relationship between stress and emotional distress, and it has been found that the frequency with which caregiving situations occur, as well as age, have predictive value on the levels of emotional distress of primary caregivers. Regarding age, we have found similar results to the literature [18], where lower ages have been associated with higher stress scores. A difference has been observed in terms of pathology-specific stress level, with the caregivers of paediatric patients being those who have presented the highest levels, followed by respiratory problems and finally SS.

Regarding the limitations of the study, results must be considered with care when attempting to apply them more generally, since our sample is non-probabilistic, and only parents living in the Valencian Community have participated. Because mothers usually take on the role of primary caregiver [61], the majority of our sample are women, which has meant that the sample cannot be evenly distributed by sex as the percentage of men was low. In this study we have considered three of the most prevalent pathologies in Spain, but we have not analysed other pathologies in which parents derive stress from caregiving and are relevant to the current landscape, as is the case of childhood cancer. On the other hand, the data from the study came from measurements obtained only through questionnaires, without obtaining measurements through other complementary means. Finally, PIP measures have been linked to those of anxiety and depression because when it comes to the consequences of the stress felt by these parents in regards to care, anxiety and depression are associated most often [7–9], but we have not considered other relevant pathologies that may result as a consequence of this stress, such as post-traumatic stress disorder [62,63]. Future directions of the brief PIP measure must consider a larger sample, including samples from other Spanish-speaking countries, in order to be able to compare them. They must also try to expand the number of men in the sample so that equitable groups can be stablished, and carry out a non-probabilistic sampling. For this purpose, it would also be convenient to increase the number of paediatric chronic pathologies that are considered in the study. In addition to all of this, it would be interesting to combine the measures taken through the questionnaires with other objective, physiological, or even hetero-administration measures, thus helping to improve inter-observer reliability [64]. It would behoove us to obtain more measures in addition to anxiety and depression, as there are more pathologies associated with the adjustment to the disease of these primary caregivers. Another objective that needs to be met, given the results obtained, will be to re-administer the reduced version of the PIP to new participants for the sake of validating the new mean and calculating the percentiles in order to facilitate the interpretation of the results in clinical practice. It will also be important to introduce the use of this questionnaire into daily clinical practice, so that physicians can detect the emotional complications that parents may develop from caring for their children's illness early on.

Despite these limitations, this study seems to make an important contribution to the field. First, this study offers a reduced version of the PIP, making it easier to complete. This will promote its application, and the results obtained through it will be more accurate and reliable, given that there will be no biases derived from the fatigue that can result from answering a long questionnaire. Shortening the questionnaire will have benefits both in the area of research (as it will leave room for further action) and in clinical practice (in a short period of time the physician will be able to assess the status of the parents and act as circumstances require). The previous study in the Spanish population [35], in which the instrument was adapted and translated, did not observe its psychometric properties in the Spanish context by means of robust methodologies. This is a limitation that we have solved in this paper, thus justifying its use in Spain. In addition, in this study, the three pathologies with the highest prevalence in Spain have been used and compared with each other, which allows conclusions to be drawn that are not possible when the studies only use them separately. The predictive capacity of PIP with respect to anxiety and depression has been demonstrated, and these are the most common conditions in these caregivers. This is relevant because they can affect the quality of life and well-being of caregivers, can lead to other pathologies in the caregiver, can worsen their health [12], can deteriorate their children's adjustment to the disease [11] and, as a consequence of all of the above, can lead to increased health expenditure [1–3]. According to the World Health Organization (WHO), health is the interaction of multiple social, political, economic, cultural, and scientific factors, making this concept a complex reality that requires an interdisciplinary approach to address [65]. Therefore, it is especially important to establish research areas that detect and try to meet the demands of health professionals, patients and their families as a whole, in order to meet their needs and thus promote quality care in the health system.

For these reasons, this brief version of the PIP is important because it will contribute to optimizing the development of specialized interventions that seek to alleviate the overload on the primary caregiver and, indirectly, on the paediatric patient. Reducing the questionnaire will help to speed up health care and the resulting interventions will facilitate the improvement of both the family and the health system, reducing the negative impact that chronic paediatric illnesses can have on the financial, social, emotional, and health problems that overburden the caregiver.

In summary, the brief version of the PIP for a Spanish context has sufficient empirical support to be considered a valid and useful instrument for the evaluation of parental stress in these difficult circumstances, by revealing the levels of distress which these carers are suffering from and thus offering an opportunity to plan interventions aimed at this population.

## Supporting information

S1 Dataset Data set complete. (DAT)

S2 Dataset Data set EFA. (DAT)

S3 Dataset Data set CFA. (DAT)
